# Efficacy and safety of midazolam combined with dezocine for sedation and analgesia in digestive endoscopy: A prospective open single-center study

**DOI:** 10.3389/fphar.2022.945597

**Published:** 2022-11-03

**Authors:** Yongpeng Chen, Jiachen Sun, Yi Lu, Liping Fu, Xueyuan Xiang, Yanan Liu, Xianhua Zhuo, Mirigul Kurban, Chujun Li

**Affiliations:** ^1^ Department of Gastrointestinal Endoscopy, The Sixth Affiliated Hospital, Sun Yat-sen University, Guangzhou, Guangdong, China; ^2^ Guangdong Provincial Key Laboratory of Colorectal and Pelvic Floor Diseases, The Sixth Affiliated Hospital, Sun Yat-sen University, Guangzhou, Guangdong, China; ^3^ Department of Otolaryngology Head and Neck Surgery, Sun Yat-Sen Memorial Hospital of Sun Yat-Sen University, Sun Yat-sen University, Guangzhou, Guangdong, China; ^4^ Department of Small Bowel Endoscopy, The Sixth Affiliated Hospital, Sun Yat-sen University, Guangzhou, Guangdong, China

**Keywords:** midazolam, digestive endoscopy, sedation, analgesia, dezocine

## Abstract

**Objective:** Digestive endoscopy is an important means of diagnosing and treating gastrointestinal diseases and a tool for screening and monitoring early gastrointestinal tumors. Digestive endoscopy can be performed using midazolam combined with dezocine for sedation and analgesia. This study explored the efficacy and safety of midazolam combined with dezocine.

**Methods:** A total of 135 patients undergoing digestive endoscopy in the Department of Gastrointestinal Endoscopy of the Sixth Affiliated Hospital, Sun Yat-sen University, from June 2021 to September 2021, were enrolled and non-blindly and non-randomly divided into a sedation-endoscopy-group (SEG, *n* = 45), anesthesia-endoscopy-group (AEG, *n* = 44), and ordinary-endoscopy-group (OEG, *n* = 46). Vital signs, levels of sedation and analgesia, the degree of pain during colonoscopy, satisfaction, and the incidence of complications were compared among the three groups.

**Results:** There were no statistically significant differences in vital signs (blood pressure, pulse, respiration, and blood oxygen saturation) among the three groups before endoscopy (*p >* 0.05). The AEG reported no pain during colonoscopy, and the pain score during colonoscopy for the SEG was lower than that for the OEG (1.11 ± 1.21 *vs*. 3.00 ± 1.16, *p <* 0.001). The scores for satisfaction were 8.84 ± 1.30 points in the SEG, 8.95 ± 1.10 points in the AEG, and 6.37 ± 0.90 points in the OEG; the differences were statistically significant (*p <* 0.001). The total incidence of complications in the AEG was 38.64% (17/44), which was significantly higher than that in the SEG [13.33% (6/45)] and OEG [13.04% (6/46)] (*p <* 0.001). In the SEG, the overall incidence of complications in women was significantly higher than that in men (*p =* 0.027).

**Conclusion:** Digestive endoscopy using midazolam combined with dezocine for sedation makes patients more comfortable, more satisfied and more compliant than the ordinary endoscopy. Additionally, it is comparable to endoscopy under general anesthesia with propofol with regard to comfort, satisfaction, and patient compliance and comparable to the ordinary endoscopy with regard to safety. Considering the shortage of anesthesiologists, the application of midazolam combined with dezocine in digestive endoscopy is worthy of clinical popularization. This study has been registered in the Hospital Ethics Committee of the Sun Yat-sen University Sixth Affiliated Hospital (Ethical Number: 2021ZSLYEC-182).

## Introduction

Digestive endoscopy is used for the diagnosis and treatment of gastrointestinal diseases and for the screening and monitoring of early gastrointestinal tumors. However, some patients refuse to undergo digestive endoscopy due to fear of and discomfort during the operation (Siegel et al., 2012). Colonoscopy is considered the gold standard examination for screening colorectal polyps and digestive tract cancers, and there is evidence that its use reduces the mortality rate of colorectal cancer (Lim et al., 2019). Some patients experience stress, nausea, vomiting, bucking and pain during digestive endoscopy (Xiao et al., 2016), interrupting examinations and treatments, affecting examination quality, and potentially worsening the condition, resulting in critical illness (Shen et al., 2015). Sedation and analgesia are considered key components because they can reduce anxiety and discomfort, thereby improving surgical tolerance and patient satisfaction, reducing the risk of complications, and improving conditions for examination (das Neves et al., 2016). In addition, appropriate sedation can improve the quality and performance of colonoscopy and the detection rate of colonic polyps (Lim et al., 2019). Moreover, sedation can increase the discovery rate of advanced lesions, which are precursors to colon cancer, by 25% in deeply sedated patients (Lim et al., 2019).

Intravenous propofol anesthesia can successfully alleviate patient anxiety and improve patient comfort during digestive endoscopy, consequently increasing patient acceptance and tolerance (Adams et al., 2017). However, the depth of intravenous anesthesia varies significantly across individuals, dosages and interval times, and cardiopulmonary dysfunction can occur, necessitating constant monitoring. Anesthesia must be administered by experienced professionals who have obtained airway management training, and this requirement increases labor costs (das Neves et al., 2016; Jin et al., 2017). Digestive endoscopy with sedation and analgesia has been shown to effectively reduce patient anxiety, increase patient acceptance, relieve intraoperative discomfort, and increase tolerance and satisfaction, all of which contribute to a high-quality examination. As a result, sedation and analgesia have been gradually implemented in clinical practice in recent years (das Neves et al., 2016; Childers et al., 2015). Sedation and analgesia drugs can be provided by nurses through the guidance of a digestive endoscopist. Benzodiazepines in combination with opioids are still utilized in most countries (Dossa et al., 2021), however, the medication regimens, dosages and times remain unstandardized.

Midazolam, a benzodiazepine with rapid onset, a short elimination half-life, small local irritation, a wide safety limit, a high therapeutic index, antianxiety effects, the ability to induce anterograde amnesia, and stable hemodynamics and without accumulation and residual effects, has been widely used for sedation, but its analgesic effect is unsatisfactory (Lee et al., 2021). One study showed that compared with gastroscopy performed under anesthesia using only propofol and fentanyl, the amount of propofol decreased and the satisfaction of patients increased after the addition of midazolam (das Neves et al., 2016). Many international guidelines, such as the German Society for Gastroenterology, Digestive and Metabolic Diseases (GSGMD) and the Spanish Society of Gastrointestinal Endoscopy (SSGE), recommend midazolam as the first-line benzodiazepine drug for sedation induction in digestive endoscopy (Igea et al., 2014; Riphaus et al., 2016).

Dezocine is an opioid analgesic with minimal addiction potential, that is, frequently used in digestive endoscopy (Xu et al., 2016). One study showed that compared with fentanyl and propofol, the combination of dezocine and propofol for colonoscopy could improve the safety of surgery and reduce the occurrence of adverse reactions (Xu et al., 2016). Another study showed that propofol combined with dezocine could reduce the dosage of propofol, reduce the risk of inhibiting the cardiovascular and respiratory systems, increase the analgesic effect, reduce limb activity, shorten the recovery time, and improve the quality of recovery (Li et al., 2019) Therefore, we aimed to investigate the efficacy and safety of midazolam combined with dezocine in digestive endoscopy through a prospective study.

## Methods

**FIGURE 1:**
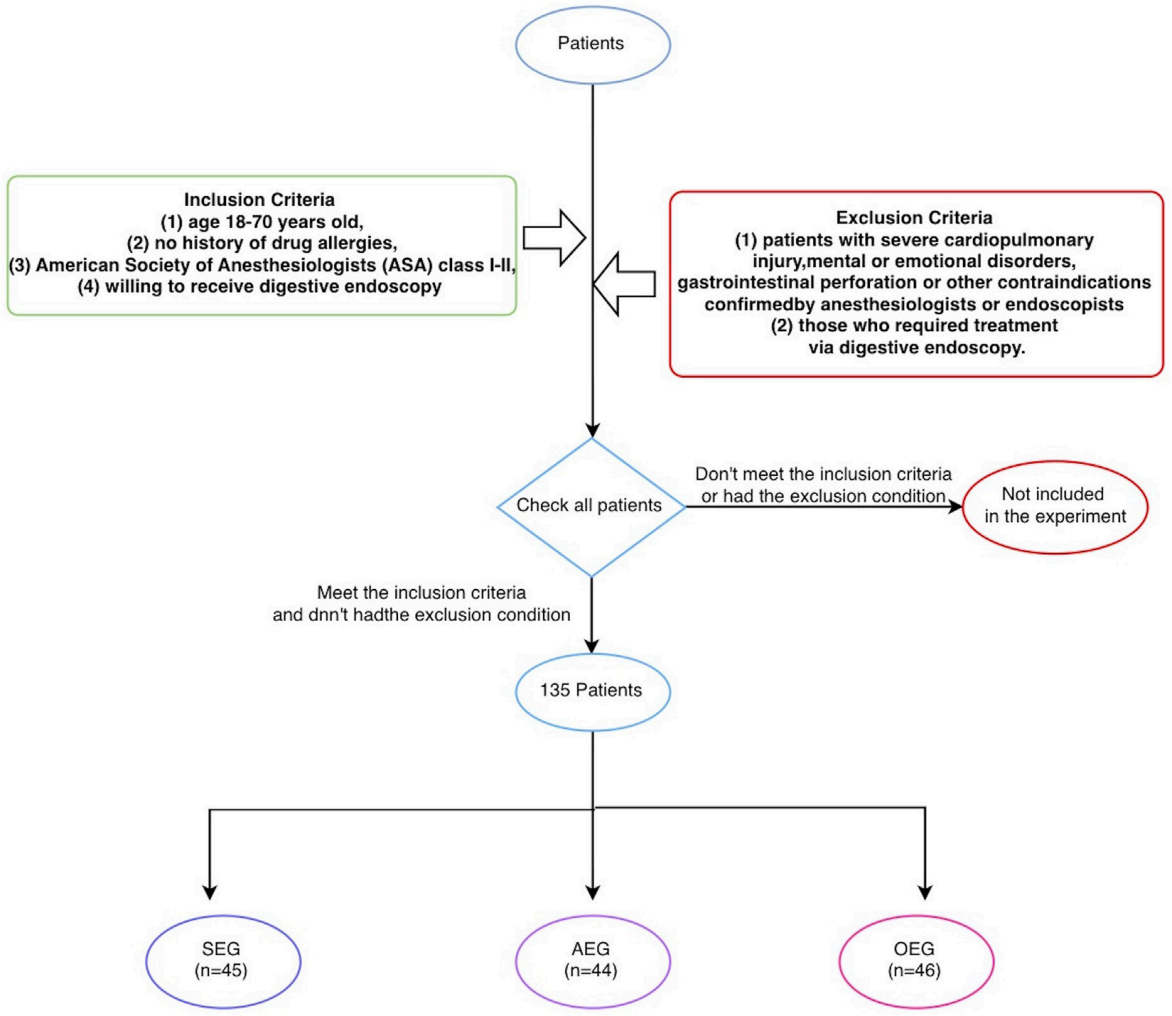
The process of grouping patients.

1) Subjects: Patients treated in the Department of Gastrointestinal Endoscopy of the Sixth Affiliated Hospital, Sun Yat-sen University, from June 2021 to September 2021 were selected. The inclusion criteria were as follows (Siegel et al., 2012): age 18–70 years old (Lim et al., 2019), no history of drug allergies (Xiao et al., 2016), American Society of Anesthesiologists (ASA) class I-II, and (Shen et al., 2015) willing to receive digestive endoscopy. The exclusion criteria were as follows (Siegel et al., 2012): patients with severe cardiopulmonary injury, mental or emotional disorders, gastrointestinal perforation, or other contraindications confirmed by anesthesiologists or endoscopists and (Lim et al., 2019) those who required treatment *via* digestive endoscopy. The patients were non-blindly and non-randomly divided by the digestive endoscopist into a sedation endoscopy group (SEG), anesthesia endoscopy group (AEG), and ordinary endoscopy group (OEG). All digestive endoscopy procedures were performed by the same doctor, who had substantial experience in performing such examinations (>10,000 cases). This study was approved by the Hospital Ethics Committee (Ethical Number: 2021ZSLYEC-182), and written informed consent was obtained from all patients (as Figure 1).
2) Preoperative preparation: All patients received educational information and a bowel preparation protocol, and they were deprived of water for at least 2 h and food for at least 6 h before the procedure. All patients were assessed jointly by anesthesiologists and the endoscopist on the day of the examination, were informed of precautions, and were cooperative. Before the examination, a vial of lidocaine mucilage was sublingually administered for more than 1 min to achieve local oropharyngeal anesthesia and remove gas bubbles in the stomach.
3) Methods of sedation, analgesia and anesthesia: The depth of sedation was assessed using the Ramsay sedation scale (Table 1) (Rudner et al., 2003); patients receiving sedation and analgesia were expected to reach level III-IV, and those receiving anesthesia were expected to reach level V-VI. Patients in the SEG were intravenously injected with midazolam (NMPN H10980025, 0.02–0.05 mg/kg) and dezocine (NMPN H20080329, 0.05 mg/kg), and additional doses of midazolam and dezocine were administered according to intraoperative pain and vital signs. Patients in the AEG were intravenously injected with propofol (1.0–2.5 mg/kg) to induce anesthesia; the endoscope was inserted when the eyelash reflex disappeared, and the depth of anesthesia was maintained during the procedure. Supplemental oxygen was provided to all patients through a nasal cannula (3 L/min) throughout the examination, and the mean arterial pressure (MAP), pulse rate (PR), respiratory rate (RR) and blood oxygen saturation (SpO_2_) were recorded using an automatic monitor.
4) Data recording: All patients underwent gastroscopy before colonoscopy. During the examination, the MAP, PR, RR, and SpO_2_ were continuously monitored and recorded at seven time points: T1, before gastroscopy; T2, 3 min after beginning gastroscopy; T3, at the end of gastroscopy; T4, before colonoscopy; T5, when reaching the ileocecal valve; T6, 5 min after beginning colonoscopy; and T7, at the end of colonoscopy. The blood pressure was recorded as mild (increase or decrease in MAP < 20%), moderate (increase or decrease in MAP within 20%–40%) or severe (increase or decrease in MAP > 40%) adverse fluctuations. A peripheral SpO_2_ value less than 85% was defined as hypoxemia (Wu et al., 2014). Patient satisfaction with the digestive endoscopy and level of sedation, the nausea/vomiting/dizziness score, and whether the patient would choose this procedure again or recommend this examination method were assessed postoperatively using questionnaires. An 11-point numerical rating scale (NRS) was used for subjective scoring, where 0 points indicated “very dissatisfied” and 10 points indicated “very satisfied.” The complications included severe blood pressure fluctuations, hypoxemia, nausea, vomiting, bucking, intestinal mucosal injury, and pain during the examination.
5) SPSS 21.0 software was used for the statistical analysis. All data were expressed as percentages, means and standard deviations; or medians and interquartile ranges as appropriate. Measurement data were expressed as (‾χ±s); Comparisons of baseline and clinical characteristics among groups were analyzed by one-way ANOVA or Kruskal-Wallis one-way ANOVA, and pairwise comparisons within groups were conducted using the LSD test or paired test. Enumeration data were expressed as rates (%); differences among groups were analyzed using the chi-square test, and pairwise comparisons within groups were conducted using the Bonferroni test. All statistical tests were 2-sided, and a *p* < 0.05 was considered significant.

**TABLE 1:** Ramsay scale for the level of sedation and analgesia (Rudner et al., 2003).

Description	Score
Patient paralyzed, unable to assess level of sedation	0
Patient anxious, agitated, or restless	I
Patient cooperative, oriented, and tranquil	II
Patient sedated but responds to commands	III
Patient asleep but responds to glabellar tap	IV
Patient asleep but responds to nail bed pressure (no response to glabellar tap)	V
Patient asleep, no response to nail bed pressure	VI

## Results

### General data

A total of 135 patients [70 males (51.85%)] with an average age of 51.60 ± 13.01 years and a body mass index (BMI) of 23.44 ± 3.03 were enrolled in this study. The basic clinical characteristics of the enrolled patients are provided in Table 2.

**TABLE 2:** Basic clinical characteristics of the enrolled patients.

	SEG (*n* = 45)	AEG (*n* = 44)	OEG (*n* = 46)	Statistics	*p*
Sex				5.002	0.082
Male	20	20	30		
Female	25	24	16		
Age	52.86 ± 10.86	51.75 ± 12.60	50.26 ± 15.24	0.149	0.928
BMI	23.22 ± 3.23	23.28 ± 2.46	23.82 ± 3.03	0.533	0.588
Education level				7.331	0.119
Junior high school and below	15	12	13		
Senior high school	6	8	16		
University and above	24	24	17		
Smoking history				2.795	0.247
Yes	5	2	7		
No	40	42	39		
Drinking history				2.703	0.259
Yes	5	1	4		
No	40	43	42		
History of gastrointestinal surgery				5.307	0.070
Yes	8	3	2		
No	37	41	44		
History of gastrointestinal endoscopy				3.844	0.146
Yes	36	39	43		
No	9	5	3		
Family history of tumors				0.142	0.932
Yes	5	6	6		
No	40	38	40		

Sedation-endoscopy-group (SEG), anesthesia-endoscopy-group (AEG), and ordinary-endoscopy-group (OEG).

### Safety

**FIGURE 2:**
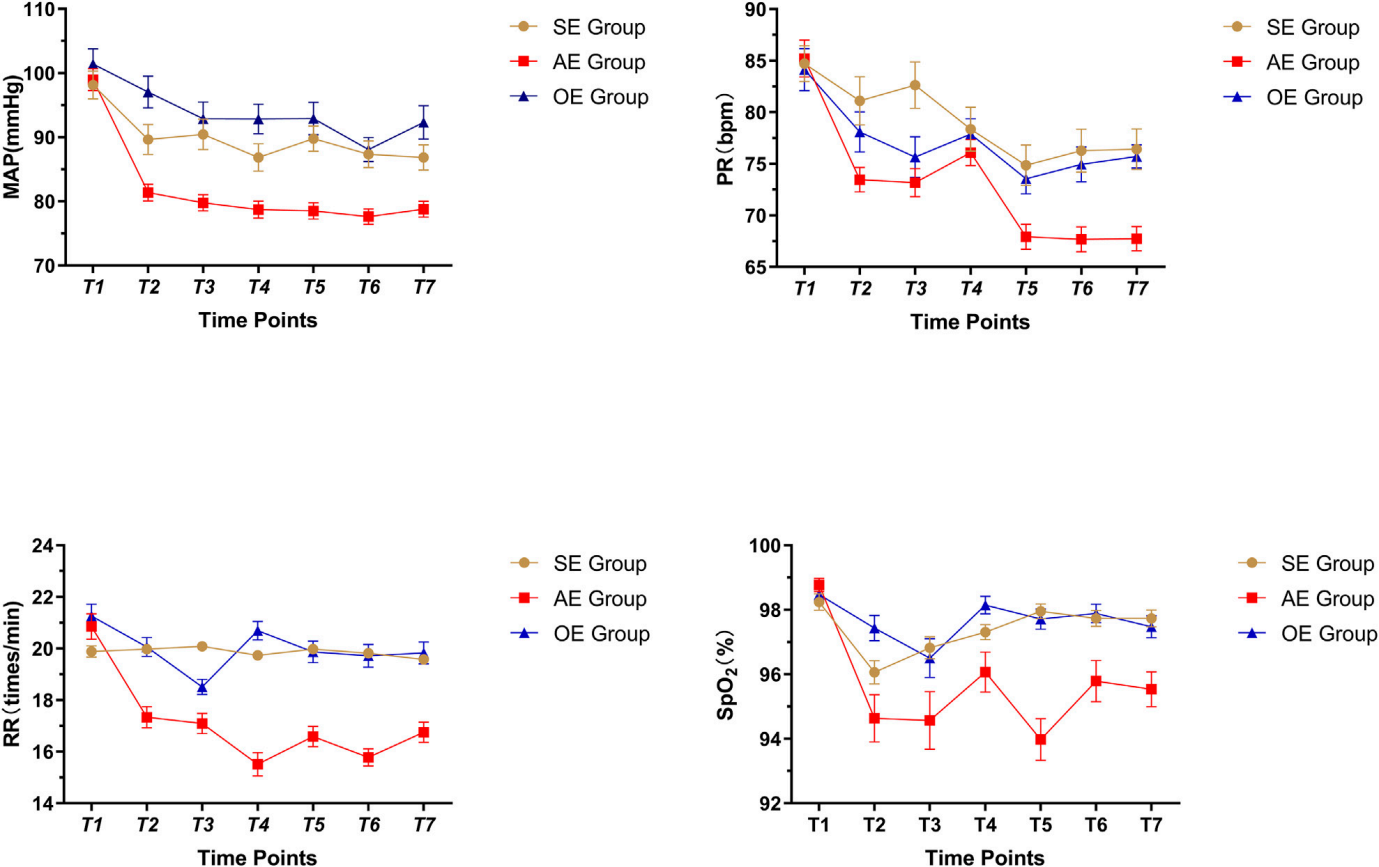
Comparison of vital signs in three groups, SE: sedation endoscopy, AE: anesthesia endocopy and OE: ordinary endoscopy.

The MAP, PR, RR, and SpO_2_ were not significantly different among the three groups before gastroscopy (*p >* 0.05). At the different time points, the MAP, PR, RR, and SpO_2_ were all within normal ranges; however, there were statistically significant differences among the groups (*p <* 0.05) (as Figure 2).

Vital signs were recorded at seven different time points. No statistically significant differences were found among groups at T1. At T7, the vital signs in the AEG were significantly lower than those in the SEG and OEG; there were no statistically significant differences between the SEG and OEG.

**FIGURE 3:**
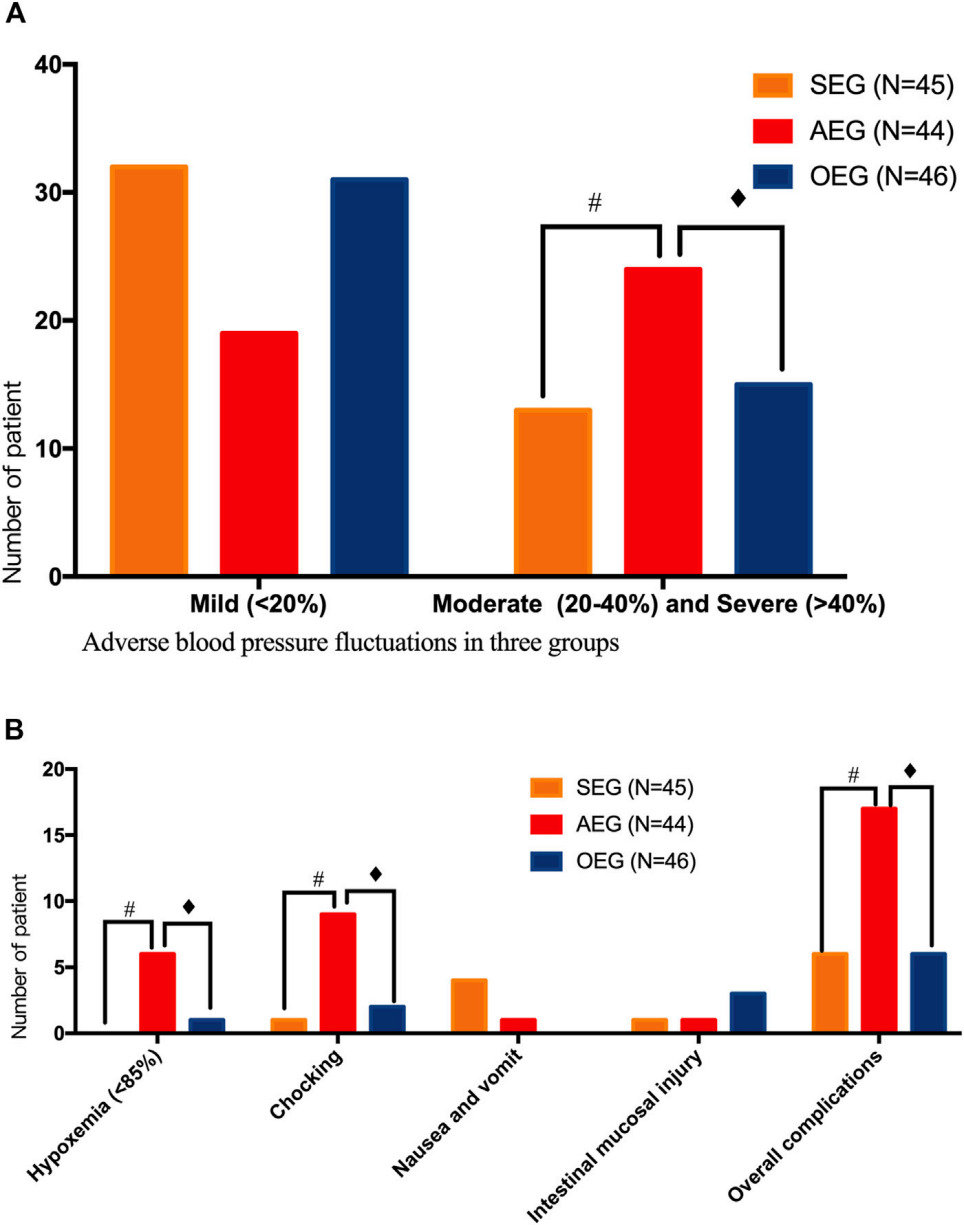
Safety assessment of gastrointestinal endoscopy in the different groups sedation-endoscopy-group (SEG), anesthesia-endoscopy-group (AEG), and ordinary-endoscopy-group (OEG). # Statistically significant difference between the SEG and AEG (*p* < 0.05). ♦ Statistically significant difference between the OEG and AEG (*p* < 0.05). **(A)** Comparison of adverse blood pressure fluctuations in three groups;**(B)** Comparison of other adverse reactions and complications in three groups.

The sedation score was 2.35 ± 1.10 points in the SEG, which was significantly lower than that in the AEG but significantly higher than that in the OEG (*p <* 0.05). The overall complication rates were 13.33% (6/45), 38.64% (17/44), and 13.04% (6/46) in the SEG, AEG, and OEG, respectively, and the differences were statistically significant (*p <* 0.05). Based on the results of pairwise comparisons, the overall incidence of complications in the AEG was significantly higher than that in the SEG and OEG (*p <* 0.05). The incidence of moderate or greater blood pressure fluctuations was 28.89% (13/45), 55.81% (24/43), and 32.61% (15/46) in the SEG, AEG and OEG, respectively; the differences were statistically significant (*p <* 0.05). Furthermore, the AEG had a significantly higher proportion of patients with moderate or greater blood pressure fluctuation than did the other two groups (*p <* 0.05). Moreover, the incidence of hypoxemia was 0, 13.95% (6/43), and 2.17% (1/46) in the SEG, AEG, and OEG, respectively; the differences were statistically significant (*p <* 0.05). The incidence of hypoxemia was significantly higher in the AEG than that in the other two groups (*p <* 0.05) (Figure 3).

Complications occurred in six patients in the SEG, including four cases of nausea and vomiting during diagnosis and treatment, one case of bucking during gastroscopy, one case of frictional injury to the intestinal mucosa, and one case of severe pain. For those who could not tolerate colonoscopy, midazolam (2 mg iv) + dezocine (2.5 mg 4) were administered in addition to the initial doses [midazolam (3 mg iv) + dezocine (2.5 mg iv)]. In the SEG, the total incidence of complications in women was significantly higher than that in men (*p <* 0.05).Age, history of surgery, smoking, drinking, previous gastrointestinal endoscopy, and family history of tumor are not related factors for complications in the SEG. (Table 3).

**TABLE 3:** Factors related to complications in the SEG (Fisher’s exact probability test).

	Complications (*n* = 6)	No complications (*n* = 39)	*p*
Sex			0.027
Male	0	20	
Female	6	19	
Age (Y)			0.385
<50	1	16	
≥50	5	23	
Medical history			0.396
No	2	22	
Yes	4	17	
History of surgery			1.000
No	4	24	
Yes	2	15	
Smoking history			1.000
No	6	34	
Yes	0	5	
Drinking history			1.000
No	6	34	
Yes	0	5	
History of gastroscopy			1.000
No	1	8	
Yes	5	31	
Family history of tumors			0.125
No	4	36	
Yes	2	3	
Intraoperative medication			0.665
No additional drugs	4	19	
Additional drugs	2	20	
Dosage of dezocine			0.569
<5 mg	6	32	
= 5 mg	0	7	

### Patient tolerability

The incidence of post-resuscitation amnesia was 26.67% (12/45), 43.18% (19/44), and 0 in the SEG, AEG, and OEG, respectively; the differences were statistically significant (*p <* 0.05). The four patients with total amnesia were all in the SEG. The overall nausea score in the SEG (0.51 ± 1.14) was not significantly different from that in the AEG but was significantly lower than that in the OEG (*p <* 0.05). The pain score during colonoscopy in the SEG (1.11 ± 1.21) was significantly higher than that in the AEG but significantly lower than that in the OEG (*p <* 0.05). The satisfaction score was 8.84 ± 1.30 points in the SEG, which was not different from that in the AEG but was significantly higher than that in the OEG (*p <* 0.05). The proportion of patients who would choose the method again and recommend it was 76.09% (35/46) in the OEG, which was significantly lower than that in the SEG [97.78% (44/45)] and AEG [97.73% (43/44)] (*p <* 0.05) (Table 4).

**TABLE 4:** Comparison of patient tolerability among the different groups (‾χ±s).

	SEG (*n* = 45)	AEG (*n* = 44)	OEG (*n* = 46)	Statistics	*p*
Postresuscitation amnesia				33.692	0.001
Complete memory	33	25	44		
Partial amnesia	8	19	0		
Total amnesia	4	0	0		
Nausea score during gastroscopy	0.58 ± 0.87	0	2.77 ± 1.11	88.466	0.001*#♦
Overall nausea score	0.51 ± 1.14	0	2.63 ± 1.11	86.065	0.001*♦
Vomiting score	0.18 ± 0.61	0	2.66 ± 1.26	97.026	0.001*♦
Dizziness score	0.44 ± 0.81	1.64 ± 1.99	0.46 ± 0.82	18.281	0.001*♦
Pain score during colonoscopy	1.11 ± 1.21	0	3.00 ± 1.16	82.974	0.001*#♦
Overall pain score	0.40 ± 0.78	0	3.00 ± 1.16	95.543	0.001*♦
Patient satisfaction	8.84 ± 1.30	8.95 ± 1.10	6.37 ± 0.90	78.888	0.001*♦
Choose again	44	43	35	16.358	0.001*♦
Recommend to others	44	43	35	16.358	0.001*♦

Sedation-endoscopy-group (SEG), anesthesia-endoscopy-group (AEG), and ordinary-endoscopy-group (OEG).

*Statistically significant difference between the SEG and OEG (*p* < 0.05); # Statistically significant difference between the SEG and AEG (*p* < 0.05); ♦ Statistically significant difference between the OEG and AEG (*p* < 0.05).

## Discussion

Demands for screening and patient acceptance of digestive endoscopy have gradually increased as public awareness of health has improved. Hence, endoscopists’ workloads have increased, as have the requirements for comfortable and efficient examinations (Dossa et al., 2021).

Appropriate sedation can help alleviate discomfort during endoscopy, thus improving the tolerance of and acceptance by patients and increasing the success rate of examinations. In contrast, poor sedation may cause insufficient cooperation by patients or fear resulting from stress-related discomfort or injury, leading to poor satisfaction and compliance (El Shahawy and El-Fayoumy, 2019). Because the requirements for comfort during digestive endoscopy have increased, sedation quality has become a principal indicator for measuring satisfaction, and patient satisfaction with the procedure directly reflects the sedative effect and sedative quality (Kilgert et al., 2014). Both the European Society of Gastrointestinal Endoscopy (ESGE) (Dumonceau et al., 2015) and SSGE(11) have noted that moderate sedation is conducive to achieving favorable satisfaction with digestive endoscopy. Another study revealed that moderate sedation not only elevates patient satisfaction with digestive endoscopy but also improves patient compliance with repeated examinations (Loftus et al., 2013). A study conducted by Jin et al. (2017) noted that for patients receiving sedative and analgesic medication for digestive endoscopy, satisfaction can also be increased through midazolam sedation and active monitoring, even if there are frequent biopsies and procedures or a longer operation time.

Intravenous anesthesia with propofol greatly facilitates patient acceptance of digestive endoscopy, but relevant complications have also attracted extensive attention (Kim et al., 2020). Complications such as hypoxia, respiratory depression, apnea, hypotension and arrhythmia are closely associated with sedation depth (Lim et al., 2019; das Neves et al., 2016). Propofol has a narrow treatment window, with the potential to cause fluctuations in sedation depth, which can be difficult to manage even by professional physicians who have airway management training and possess clinical experience. Aguero *et al.* reported that hypotension and bradycardia frequently occurred in patients who were administered high-dose propofol; these effects were attributed to myocardial suppression and the interaction between propofol and muscarinic cholinergic receptor in a concentration-dependent manner (das Neves et al., 2016). Cardiovascular and cerebrovascular diseases (CCDs) are thought to be potential factors associated with endoscopy-related death. Kim *et al.* reported that age older than 70 years and endoscopy under general anesthesia were independent risk factors for the occurrence of CCD (21). In situations where endoscopy under general anesthesia is vigorously promoted, there may be a corresponding overuse of intravenous anesthesia with propofol and a waste of resources (Edelson et al., 2020). Lin *et al.* found that endoscopic procedures could be completed by inducing III-IV sedation in most patients under intravenous anesthesia with propofol rather than unconscious deep anesthesia (Lin, 2017).

Oxygen saturation tends to be decreased in elderly patients who receive colonoscopy under intravenous anesthesia with propofol (Martínez et al., 2011). Similar findings were reported in another study carried out with patients undergoing gastric submucosal dissection *via* endoscopy under general anesthesia; the results indicated that anesthesia can be induced by low-dose propofol in patients older than 80 years of age but that these patients have a higher incidence of hypoxemia than do young patients (Gotoda et al., 2016). In this study, the incidence rates of overall complications and hypoxemia in the AEG were 38.64% and 13.64%, respectively, which were much higher than those in the SEG and OEG, consistent with literature reports. Notably, the proportion of bucking in the AEG was 20.45%; this effect is correlated with not only the insufficient depth of propofol anesthesia but also the timing and technique of endoscope insertion by endoscopists.

Compared with propofol-induced deep sedation, the sedation and analgesia approach generated lower incidences of hypoxemia and overall complications, and patients were able to respond to oral commands independently or with slight tactile stimulation (Kang et al., 2016; Lin, 2017; Lim et al., 2019). Registered nurses who have been systemically trained in digestive endoscopy should assist endoscopists in sedative and analgesic endoscopic procedures, vital sign monitoring and temporary emergency treatment; such assistance can save equipment and labor costs (Dossa et al., 2021).

Benzodiazepines combined with opioids are mainly utilized for sedation and analgesia. Establishing an optimal regimen contributes to safe and efficient endoscopic operations. The American Society of Gastrointestinal Endoscopy (ASGE) and Canadian Association of Gastroenterologists (CAG) have noted that the combination of benzodiazepines and opioids is adequate for conventional digestive endoscopy, particularly when applied for the induction of moderate sedation and analgesia during colonoscopy (Keeffe, 1995; Byrne et al., 2008; Early et al., 2018; El Shahawy and El-Fayoumy, 2019). Based on systematic evaluations of cohort studies, midazolam is recommended by both the GSGMD and the SSGE as a benzodiazepine drug preferred for the induction of sedation (Igea et al., 2014; Riphaus et al., 2016). The results from a prospective trial carried out by Christe et al. (2000) indicated that midazolam reduced the MAP of elderly patients by 10 mmHg on average and that there were no severe blood pressure fluctuations. Midazolam has a prominent sedative effect and is safe, but its analgesic effect is not ideal. Dezocine combined with propofol has a notable anesthetic effect in colonoscopy performed under anesthesia, reduces the occurrence of adverse reactions, and thus is highly safe for surgery (Xu et al., 2016). In a study conducted by Baykal *et al.*, patients who underwent colonoscopy were assigned to two groups, namely, an experimental group (dezocine combined with propofol) and a control group (fentanyl combined with propofol), and the incidence of intraoperative and postoperative adverse reactions was distinctly lower and the recovery time was faster in the experimental group (Baykal Tutal et al., 2016). Li et al. (2019) reported that analgesics combined with dezocine allowed a dose reduction in analgesics, decreased the risk of cardiovascular and respiratory suppression, facilitated analgesic effects, diminished limb activity, shortened the recovery time, and improved the quality of recovery. Consistent with the above research results, favorable effects and fewer complications were obtained in this study by applying midazolam combined with dezocine to induce sedation and analgesia during digestive endoscopy. However, there is no consensus of the optimal approach and dose for the induction of analgesia and sedation in gastroscopy and colonoscopy. Therefore, numerous studies are needed to further guide more detailed and effective sedation and analgesia regimens.

Zhao et al. (2020) reported that being female was a risk factor for noncompletion of colonoscopy (OR: 1.525, 95% CI: 1.278-1.819; *p* < 0.001) and therefore that sedative colonoscopy is preferentially utilized in women (OR: 1.279, 95% CI: 1.223-1.338; *p* < 0.001). Moreover, compared with male and elderly patients (>50 years old), female and young patients are less satisfied with sedative effects, with sedatives needing to be changed frequently and more sedatives needed to achieve similar effects (Childers et al., 2015; Shingina et al., 2016; Jin et al., 2017). The results of this study indicated that more complications, especially nausea and vomiting, occurred in women during examinations under sedation and analgesia; this finding may be correlated with high anxiety levels in women. Further studies are also needed to search for the causes of adverse drug reactions (Rees et al., 2016).

There are some limitations of this study. 1) The response rate for telephone or e-mail follow-up was lower than that of on-site follow-up (Lin et al., 2007; Jin et al., 2017), the questionnaire was completed postoperatively, and the responses may have potentially been influenced by sedative and analgesic agents, inaccurate answers to all questions, or hesitation to present feelings of dissatisfaction in the presence of the data collector (Jin et al., 2017). 2) Our study was non-randomized and non-blind and it is hoped that randomized trials can be carried out in the future. 3) This was a single-center study with a small sample size. There was no definite optimal approach and dose for the induction of analgesia and sedation for gastroscopy and colonoscopy, and analyses of related factors and causes of overall complications of sedation and analgesia were insufficient; therefore, large-scale multicenter studies are needed.

In conclusion, the digestive endoscopy using midazolam combined with dezocine for sedation makes patients more comfortable, more satisfied and more compliant than the ordinary endoscopy. Additionally, it is comparable to endoscopy under general anesthesia with propofol with regard to comfort, satisfaction, and patient compliance and comparable to the ordinary endoscopy with regard to safety. Considering the shortage of anesthesiologists, the application of midazolam combined with dezocine in digestive endoscopy is worthy of clinical popularization. However, as we said above, further experiments are needed.

## Data Availability

The original contributions presented in the study are included in the article/supplementary materials, further inquiries can be directed to the corresponding author.
